# A rare case of infectious keratitis that developed 27-years after radial keratotomy

**DOI:** 10.1016/j.ajoc.2021.101240

**Published:** 2021-12-07

**Authors:** Maya Kawasaki, Hideki Fukuoka, Mariko Kawabata, Chie Sotozono

**Affiliations:** Department of Ophthalmology, Kyoto Prefectural University of Medicine, Kyoto, Japan

**Keywords:** Radial keratotomy, Late onset, Infectious keratitis

## Abstract

**Purpose:**

To report a rare case of bacterial infectious keratitis that developed 27 years after radial keratotomy.

**Observations:**

A 48-year-old female who underwent bilateral radial keratotomy (RK) 27-years previous presented at our department with pain and visual loss in her right eye after being diagnosed with bacterial keratitis by her primary care physician. Slit-lamp examination showed a focus at the deep layer of the cornea, endothelial plaque, and hypopyon. Treatment with topical fortified levofloxacin and cefmenoxime eye drops was initiated. However, at 2 days after the initiation of treatment, there was no improvement, so anterior chamber irrigation and a bacterial smear/culture were performed. The smear showed many gram-positive cocci, yet no organism was detected in the culture. We suspected the causative bacteria to be methicillin-resistant Staphylococcus aureus (MRSA) due to her job (i.e., nursing staff) and the treatment course. Thus, we initiated treatment with 0.5% arbekacin eye drops for the suspected MRSA keratitis, and it was effectively controlled.

**Conclusions and Importance:**

The findings in this case indicate that the incisions used for RK are delicate/fragile and can easily open doors to infection, as they remain unstable for many years post surgery.

## 1 Introduction

First introduced by Fyodorov in 1974, radial keratotomy (RK) is a type of refractive surgery used for the correction of myopia.^1^ However, it should be noted that in 1939, Sato^2^ reported the first prototype method of RK, i.e., anterior and posterior keratotomy, in an attempt to treat keratoconus, myopia, and astigmatism by making incisions in the cornea, as he was focused on the flattening of the cornea to improve myopia after the healing of acute hydrops.

Since then, RK has primarily been supplanted by the latest techniques used for refractive surgery, such as photorefractive keratectomy, laser-assisted in situ keratomileusis (LASIK), epipolis LASIK (epi-LASIK), small incision lenticule extraction (SMILE), and phakic intraocular lens implantation. However, the presumed number of patients who have previously undergone RK for the correction of myopia is over 2 million in the United States and Canada.

Here, we report a rare case of infectious keratitis that developed 27-years after RK.

## 2 Case report

A 48-year-old female who had undergone bilateral RK (i.e., 8 corneal incisions per eye) 27-years previously presented with the complaints of pain and visual loss in her right eye after being diagnosed with bacterial keratitis by her primary care physician. She was employed as a staff member at a nursing facility; i.e., an intermediary facility providing care between hospitals and homes. Her specific job entailed providing assistance to the elderly, including bathing, toileting, meals, and transfers, as well as facial, oral, skin, and hair cleaning, which put her in direct contact with those subjects. Post RK, she had not undergone any long-term follow-up examinations due to the good surgical outcome and the postoperative course being stable and satisfactory for 27 years. However, at 5 days prior to admission, she suddenly became aware of pain in her right eye, followed by redness and foreign body sensation (1 day later) and blurred vision (2 days later). Although she had been diagnosed with bacterial keratitis by her primary care physician at another clinic, she was not prescribed any eye-drop medication due to the secondary medical care receiving site being closer and easier for her to visit. Following initial examination at the secondary medical care receiving site, she was immediately referred for treatment at the emergency room of Kyoto Prefectural University of Medicine Hospital, Kyoto, Japan.

**Fig. 1:**
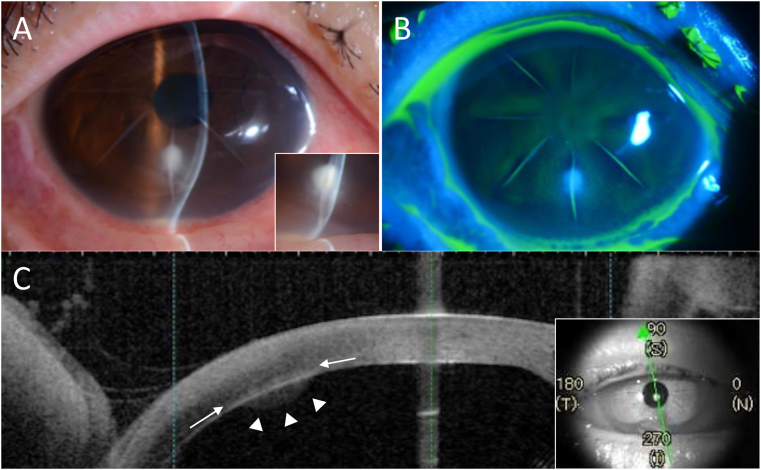
A: Slit-lamp microscopy image of the patient's right eye showing a small-sized focus with inflammatory infiltrates (1.8 × 1.3 mm) and endothelial plaque in the deep site in the radial incision at the 6-o'clock meridian, and hypopyon, and an enlargement of the image (inset). B: Image showing no epithelial defect and no aqueous humor leakage. C: Anterior-segment optical coherence tomography image showing endothelial plaque (white arrowheads), and inflammatory infiltrates (white arrow markers) located at the deep layer of the cornea (inset).

Upon presentation, it was noted that the patient had no remarkable systemic medical history, and no history of any ocular trauma or contact lens use. Initial examination of her right eye revealed a best-corrected visual acuity (VA) of 20/40. Moreover, we observed a small-sized focus area with inflammatory infiltrate (1.8 × 1.3 mm) and endothelial plaque in the deep site in the radial incision at the 6-o'clock meridian, conjunctival injection, and inflammatory cells in the anterior chamber with hypopyon (Fig. 1A). However, no epithelial defects or aqueous humor leakage were observed at that time (Fig. 1B). In the eyelids of both eyes, no blepharitis or meibomian gland dysfunction was observed. Thus, we initiated treatment with an hourly topical administration of 1.5% levofloxacin and 0.5% cefmenoxime eye drops. However, despite the treatment with broad-spectrum antibiotics, the ocular findings on the following day revealed that the conjunctival injection, the focus, and the hypopyon were worsening. Examination by anterior segment-optical coherence tomography (AS-OCT) showed a corneal plaque at the endothelial side of the cornea, as well as an elevated corneal thickness and a steepening of the inferior cornea around the focus area (Fig, 1C). Due to the fact that the levofloxacin/cefmenoxime eye-drop treatment was unsuccessful, fungal keratitis was also considered as a differential diagnosis, and natamycin ointment drops were added to the treatment.

At 2-days after the initiation of treatment, we performed irrigation of the anterior chamber and a bacterial smear/culture, using the obtained specimen for the purpose of detecting the causal pathogens and controlling the keratitis. An eye examination performed prior to the intraocular surgery showed that the corneal endothelial cell density (ECD) in her right eye and left eye was 1,346 and 1,175 cells/mm,^2^ respectively.

**Fig. 2:**
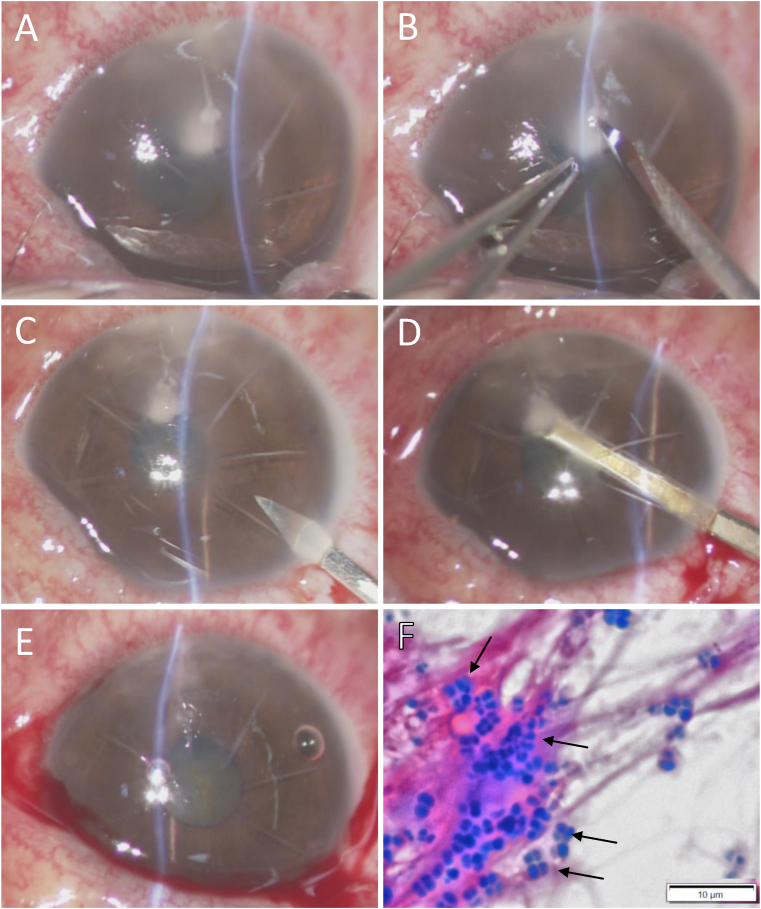
Images of the surgical steps used for collection of the specimens from around the focus area and for irrigation of the anterior chamber (A–E). Note that the images were taken from the surgeon's view. A: Image showing that the abscess had already came out after washing of the patient's right eye. B: Image obtained after making 2 side ports and an opening of the radial wound with a V-lance knife (Alcon Laboratories). C: Image showing the making of one side-port incision. D: Image showing irrigation and aspiration of the corneal plaque. E: Image obtained immediately post surgery. F: Image of the smear examination of the abscess between the radial incisions showing lots of clustered gram-positive cocci in fibrin formation (gram staining) (black arrow markers).

Since the abscess had already been extracted through the incision made at the start of the surgery, it was easy to collect specimens from the focus area. The adhesion property of the incision was found to be loose and easily separated. Thus, we made a single side-port incision with a V-lance knife (Alcon Laboratories, Inc., Fort Worth, TX) at the 10-o'clock position, followed by irrigation of the anterior chamber. The corneal plaque on the posterior surface of the cornea was then aspirated via the use of a Simcoe aspiration/irrigation cannula (Fig. 2A–E).

Laboratory examination of the obtained RK-incision specimens and anterior chamber specimen showed numerous gram-positive cocci (Fig. 2F) and only white blood cells, respectively, yet no organisms were isolated from the specimens. Considering the patient's occupation, as well as the unsuccessful initial treatment, a topical administration of 0.5% arbekacin, a select MRSA antibiotic, was initiated due to the possibility of an MRSA infection. At 3-days post treatment initiation, clinical examination revealed no hypopyon and a reduction of infiltrates.

**Fig. 3:**
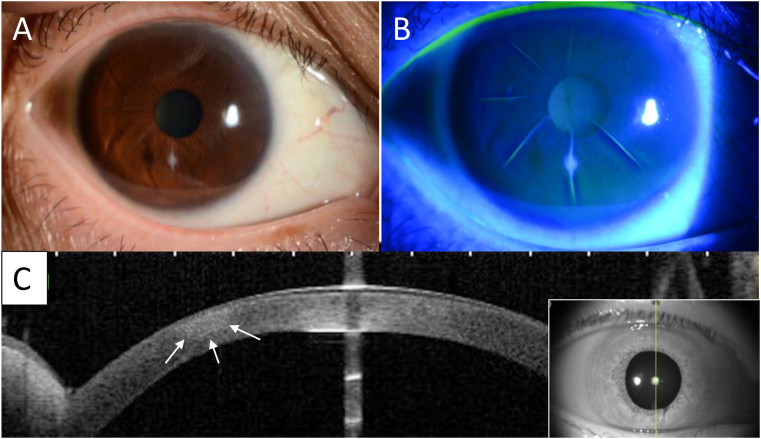
A: Slit-lamp image showing the small scar and no conjunctival injection. B: Image showing no epithelial defect. C: Anterior-segment optical coherence tomography image showing the small scar between the inferior radial incisions (white arrow markers).

Due to posterior synechia caused by the anterior chamber inflammation, tropicamide phenylephrine hydrochloride eye-drops were added to the treatment. Although the bacterial culture test was negative, the treatment was successful; i.e., the infiltrates diminished, the eye-drop treatment was gradually tapered, and the scar healed. The topical administration of the 0.5% arbekacin eye-drops was gradually tapered, and then discontinued after 1 month. At the final follow-up examination performed at 4-months after the onset of keratitis, the best-corrected VA in the patient's right eye was found to have improved to 20/20. Slit-lamp examination showed a small scar without any epithelial defect (Fig. 3A and B), and AS-OCT examination showed the small scar between the inferior radial incisions (Fig. 3C).

## 3 Discussion

Various postoperative complications after RK surgery have been reported, including pain, photophobia and glare, diurnal variation of VA, and refractive errors such as astigmatism and over- or under-correction.^3^, ^4^, ^5^ Complications that reportedly affect VA include infections such as keratitis and endophthalmitis, as well as a traumatic perforation and decreased corneal ECD.^3^ It is widely known that infections can occur at any time after surgery, i.e., from the early postoperative period to many years later.^4^^,^^6^ Moreover, *Pseudomonas aeruginosa*,^7^ staphylococci,^8^ and fungal infections have all been reported.^6^^,^^9^^,^^10^ In most cases, those infections respond well to topical antibiotic eye-drop treatment. However, when that treatment is ineffective, corneal transplantation surgery is sometimes required. Even in cases in which a drug therapy is initially successful, penetrating keratoplasty is sometimes needed in order to effectively restore vision.^10^

According to the findings in previous reports on late-onset infections post RK,^6^, ^7^, ^8^ the infections were more frequently observed in the inferior incision than in the superior. Similar to those findings, the infection in this present case occurred in the incision made at the 6-o'clock position, and at the 6- and 12-o'clock regions, the cornea is susceptible to blinks and eyelid contact. In specific, it is thought that the 6-o'clock area is an environment in which superficial punctate keratopathy is more likely to occur due to the effects of dry eye and meibomitis. Although we were unable to identify the causal pathogen in this present case, keratitis due to *Pseudomonas aeruginosa* is the infection most frequently reported.^10^ Since gram-positive cocci were detected by smear examination, and since the patient was employed as a healthcare worker, treatment was performed while keeping in mind the possibility of a resistant bacteria, such as MRSA. Since gram-positive cocci were detected in the smear test and the patient was a healthcare worker, the possibility of resistant bacteria such as MRSA was taken into consideration while treating the patient, as her occupation presented the possibility of exposure to MRSA carriage or infection^11^ and the patients she treats are at a high risk of developing MRSA keratitis. Moreover, it is important to pay additional attention to community-acquired MRSA.^12^

In this present case, keratic precipitates and hypopyon were found at the initial examination, yet no corneal defects were observed. Intraoperative findings also suggest that the incisional wound is delicate and fragile, and that the incision used for RK can remain unstable over a long-term period. Moreover, we theorize that the inflammation in the anterior chamber might have been strong, since the dissection and closure of the incision wound occurred and the bacteria directly invaded the deep corneal layer.

## 4 Conclusion

We report a rare case of infectious keratitis, a serious complication, that developed 27-years after RK. In such cases, if diagnosis and treatment are delayed, sequelae such as loss of vision can occur, thus subsequently requiring corneal transplantation to be performed in some cases. In this present case, although early detection and treatment resulted in an excellent postoperative outcome with no visual dysfunction, our findings reveal that long-term follow-up is necessary post RK since infection may occur many years after surgery.

## Patient consent

Consent to publish this case report has been obtained from the patient in writing. This case report does not contain any personal identifying information.

## Funding/support

No funding or grant support

## Authorship

All authors attest that they meet the current ICMJE criteria for Authorship.

## Declaration of competing interest

No conflicting relationship exists for any author.
